# Immediate intraoperative detrusor muscle status at primary transurethral resection of bladder tumour: a prospective, blinded, paired cohort, feasibility study using fluorescence confocal microscopy (IB1‐LaserComplete study)

**DOI:** 10.1111/bju.70395

**Published:** 2026-08-13

**Authors:** Martin J. Connor, Archana Gopalakrishnan, Nikhil Mayor, Eva Bolton, Sami Hamid, Sai Kalpitha Reddy, Tayla Perreau, Eslam Maher, Alexander Light, Samuel Morris, Jiten Jaipura, Mariana Bertoncelli Tanaka, Norma Gibbons, Archie Hughes‐Hallett, Hashim U. Ahmed, Rakesh Heer, Andrew Smith, David Hrouda, Anna Silvanto, Mona El‐Bahrawy, Mathias Winkler

**Affiliations:** ^1^ Division of Surgery, Department of Surgery and Cancer, Faculty of Medicine Imperial College London London UK; ^2^ Royal Marsden Urology The Royal Marsden Hospital, The Royal Marsden NHS Foundation Trust London UK; ^3^ Department of Urology Charing Cross Hospital, Imperial College Healthcare NHS Trust London UK; ^4^ Experimental Cancer Medicine Centre (ECMC), Department of Surgery and Cancer, Imperial College London London UK; ^5^ Department of Histopathology Imperial College Healthcare NHS Foundation Trust, Charing Cross Hospital London UK; ^6^ Department of Histopathology University College London Hospitals NHS Foundation Trust (UCLH) London UK; ^7^ Department of Metabolism, Digestion and Reproduction Imperial College London London UK

**Keywords:** bladder cancer, intraoperative pathology, fluorescence confocal microscopy (FCM), detrusor muscle, transurethral resection of bladder tumour

## Abstract

**Objectives:**

To evaluate *ex vivo* fluorescence confocal microscopy (FCM) for immediate detrusor muscle (DM) assessment during primary transurethral resection of bladder tumour (TURBT).

**Patients and Methods:**

This prospective, blinded, paired‐cohort feasibility study enrolled consecutive patients undergoing primary TURBT for suspected non‐muscle‐invasive bladder cancer between September 2024 and September 2025 (IB1‐LaserComplete study; International Standard Randomised Controlled Trial Number Register [ISRCTN]16114765). *Ex vivo* FCM generates immediate high‐resolution digital images of fresh tissue, enabling immediate DM assessment. Intraoperative *ex vivo* FCM imaging of *en face* tumour base specimens was performed using the Histolog® scanner (SamanTree Medical SA, Lausanne, Switzerland). DM status was reported by a blinded uro‐pathologist and urologist. Formalin‐fixed histopathology served as the reference standard. The primary outcome was technical feasibility, defined as acquisition of an interpretable FCM image. Secondary outcomes included exploratory diagnostic performance, inter‐observer agreement and time to confirmation of DM status.

**Results:**

A total of 34 patients were recruited and 30 had evaluable paired specimens. DM was absent in 13% (four of 30). FCM was sufficient for determining DM status in all patients (100%; 30 of 30). Sensitivity, specificity, positive predictive value, negative predictive value (NPV) were 42% (95% confidence interval [CI] 26–61%), 100% (95% CI 51–100%), 100% (95% CI 74–100%), and 21% (95% CI 9–43%), respectively. Inter‐observer agreement between the uro‐pathologist and urologist was high at 83% (κ = 0.72, 95% CI 0.47–0.97). The median (interquartile range) time to confirm DM status was 5.4 (4.95–5.39) min vs 11 (7–13.8) days using standard histopathology.

**Conclusion:**

Fluorescence confocal microscopy is feasible for rapid intraoperative imaging of TURBT specimens and demonstrates excellent specificity for detection of DM. However, its low sensitivity and NPV preclude use as a stand‐alone tool to exclude DM intraoperatively. Based on these findings, FCM should not guide intraoperative decision making outside research settings.

**Abbreviations**
DM: detrusor muscle
EAU: European Association of Urology
FCM: fluorescence confocal microscopy
ICHTB: Imperial College Healthcare Tissue Bank
IQR: interquartile range
ISRCTN: International Standard Randomised Controlled Trial Number
(N)MIBC: (non‐)muscle‐invasive bladder cancer
pT: pathological T‐stage
(N)(P)PV: (negative) (positive) predictive value
TURBT: transurethral resection of bladder tumour

## Introduction

Bladder cancer is the 10th most diagnosed cancer worldwide and is four times more common in men than in women [1]. Bladder cancer is first diagnosed by tissue obtained in surgery during a transurethral resection of bladder tumour (TURBT). Numerous studies have report an estimated 30% (15–65%) of patients do not have detrusor muscle (DM) present at primary TURBT [2, 3, 4, 5]. The absence of DM in specimens is associated with a significantly higher risk of residual disease, early recurrence, and tumour under‐staging [6, 7]. In contrast the presence of DM in the specimen is considered a surrogate measure of the ‘resection quality’ [6, 7].

Approximately 75% of patients with non‐muscle‐invasive bladder cancer (NMIBC) can have recurrence following their first TURBT [6, 7, 8]. Effective treatments for NMIBC, such as bladder instillations of BCG and mitomycin‐C, aim to reduce progression and recurrence [7, 9]. A key determinant of correct allocation of treatment at the time of TURBT is whether DM was obtained to allow accurate determination of pathological stage. The European Association of Urology (EAU) have issued strong guidance that newly‐diagnosed patients without muscle in the specimen, with the exception of Ta low‐grade/Grade 1 tumours, should undergo a second resection of tumour bearing areas to determine the exact stage and refine allocation of treatment. [1, 2] Second, resection has important negative health‐economic and quality of life implications [10, 11].

Fluorescence confocal microscopy (FCM) is a laser imaging technique producing instantaneous histological digital images of fresh tissue without the need for time‐consuming traditional fixation techniques [12]. FCM offers the opportunity to report immediate DM status intraoperatively. The technology has been successful across several different organ systems [13, 14, 15]. Within urology it has an increasing role in in real‐time intraoperative margin assessment at time of radical prostatectomy [15].

To our knowledge, no clinical trial evidence evaluating FCM in bladder cancer exists. We hypothesised that FCM could be used to accurately identify DM status at time of primary TURBT for patients with suspected NMIBC.

## Patients and Methods

### Study Design and Participants

The IB1‐LASERComplete is a prospective, blinded, paired‐cohort study conducted at a tertiary centre hospital (International Standard Randomised Controlled Trial Number [ISRCTN]16114765; Figure S1). Research ethics committee approval was given by the Health Research Authority (Wales REC3; 22/WA/0214) via the Imperial College Healthcare Tissue Bank (ICHTB). The ICHTB is supported by the National Institute for Health Research (NIHR) Biomedical Research Centre based at Imperial College Healthcare NHS Trust and Imperial College London.

The primary objective was to evaluate the feasibility of obtaining FCM images for interpretation of DM status. Secondary objectives included evaluation of the diagnostic performance of FCM for the detection of DM and inter‐observer agreement of FCM images by the uro‐pathologists and urologists.

Consecutive patients undergoing primary TURBT for suspected NMIBC were enrolled at a tertiary referral unit for bladder cancer. Patients with a radiological or clinical suspicion of MIBC were excluded. The IB1‐LASERComplete study protocol was prospectively registered (ISRCTN16114765) prior to enrolling the first patient.

### Surgical Procedure

We permit the use of piecemeal and *en bloc* resection with bipolar or monopolar diathermy as per operating surgeon standard procedure. We asked operating surgeon to provide a separate deep tumour base sample; this may have been obtained from loop resection or cold‐cup biopsy as per operating surgeon standard procedure.

### Digital Pathology Acquisition Procedure

We have previously published our embedded internal pilot of nine patients that was performed to develop the standard operating procedure (Figure S2) [16]. FCM images were acquired by the Histolog® Scanner using a fluorescent acridine orange stain (Histolog® dip) applied to the specimen (SamanTree Medical SA, Lausanne, Switzerland). A single‐face (48 × 36 mm) image with a maximum resolution of 8000 × 8000 pixels was acquired (~45 s). The deep specimen of bladder tissue from the dominant tumour was immersed for 10 s in the Histolog dip. The specimen was then rinsed with three drops of 0.9% saline. The specimen was scanned on anterior and posterior surfaces (Figure S2). Images were pseudonymised at the point of scanning. Specimens then underwent formalin fixation and paraffin embedding for standard‐of‐care histopathological assessment.

### Digital Pathology Reporting Procedure

A single blinded uro‐pathologist (A.S.) and urologist (M.J.C.) undertook confocal imaging training module developed by the device manufacturer as well as reviewing prior internal pilot study cases with histological verification prior to commencing the study. At bedside, a single urologist (M.J.C.) reported the presence of DM as ‘present’ or ‘not present’. A single uro‐pathologist (A.S.) independently reported all FCM digital images using a study specific reporting form (Data S1), blind to the final formalin‐fixed pathology report and the bedside urologist's interpretation. Formalin‐fixed pathology reporting was completed by dedicated NHS uro‐pathologists. As this was a feasibility study no repeat resection was taken if either urologist or uro‐pathologist reporting on FCM images identified the absence of DM.

### Outcomes

The primary outcome was feasibility, defined as the acquisition of an FCM image of sufficient diagnostic quality to permit uro‐pathologist interpretation of DM status at first TURBT.

Secondary outcomes included diagnostic performance of FCM for DM status on a per‐patient level (sensitivity, specificity, positive predictive value [PPV], negative PV [NPV]), and inter‐observer agreement of FCM imaged reported by the independent uro‐pathologist compared to bedside urologist.

### Statistical Analysis

Sample size was pre‐specified in the statistical analysis plan at 30 patients for the main study with a minimum internal pilot of five patients to develop the standard operating procedure (SOP). As this was a feasibility study, no specific hypothesis was powered. We estimated that 30 patients would allow us to encounter substandard processes with a high probability (97.5%) when there is a 10% chance of any occurring in a given sample [17]. It will also provide us with a range of plausible values and nuisance parameters, such as the prevalence of DM in this population, to be able to carefully plan a larger study.

Contingency tables were used to calculate sensitivity, specificity, PPV, and NPV, comparing uro‐pathologist FCM with final histopathology for the detection of DM. The inter‐observer agreement of the blinded uro‐pathologist and the bedside urologist reporting FCM was quantified using percentage agreement and Cohen's kappa index. Time to complete FCM analysis was from when specimen was positioned on the scanner and immersed in fluorescent acridine orange stain until the digital image appeared. The CIs were calculated using the Wilson method. All other analysis were exploratory. All statistical analyses were conducted using R, version 4.4.2 (R Foundation for Statistical Computing, Vienna, Austria). Reporting was in accordance with the Standards for Reporting of Diagnostic Accuracy (STARD) guidelines [18].

## Results

### Overview

Between September 2024 and 2025, 34 patients were consented to the main study. Four patients were excluded following recruitment as the deep sample was not sent separately to the remaining bladder tumour tissue for formalin‐fixed histopathological assessment (Figure S3).

### Patient and Tumour Characteristics

Overall, the median (interquartile range [IQR]) age was 70 (64–81) years, 27% (eight of 30) female and 73% (22 of 30), respectively (Table 1). Unifocal tumours were present in 67% (20 of 30), with tumours of <3 cm in 67% (20 of 30). Solid tumours were present in 40% (12 of 30) and papillary tumours in 70% (21 of 30), with three patients exhibiting both solid and papillary morphology. All cases underwent piecemeal resection without use of *en bloc* techniques.

**Table 1:** Patient, surgical and tumour characteristics.

Variable	Value
Total number of patients, *N* (%)	30 (100)
Sex, *n* (%)	
Female	8 (27)
Male	22 (73)
Age, years, median (IQR)	70 (64–81)
Ethnicity, *n* (%)	
Arab	1 (3.3)
Asian	8 (27)
Black	1 (3.3)
Mixed	1 (3.3)
Other	7 (23)
White	12 (40)
Smoker, *n* (%)	14 (47)
Past medical history, *n* (%)	14 (47)
Myocardial infarction	7 (23)
Hypertension	19 (63)
Congestive heart failure	2 (6.7)
Cardiovascular disease (including AAA)	3 (10)
Cerebrovascular accident (including TIA)	4 (13)
Dementia	1 (3.3)
Chronic lung disease	5 (17)
Diabetes, *n* (%)	
End‐organ	1 (3.3)
None/diet	22 (73)
Uncomplicated	7 (23)
Chronic kidney disease, *n* (%)	
Moderate/severe (Stage ≥3)	9 (30)
No	21 (70)
History of pelvic radiotherapy, *n* (%)	1 (3.3)
Charlson Comorbidity Index score, median (IQR)	5.50 (4.00–9.00)
Tumour focality, *n* (%)	
Multifocal	10 (33)
Unifocal	20 (67)
Tumour size, *n* (%)	
≥3 cm	10 (33)
<3 cm	20 (67)
Surgical resection, *n* (%)	
Piecemeal	30 (100)
*En bloc*	0 (0)
Diathermy, *n* (%)	
Bipolar	3 (10)
Monopolar	21 (70)
Unknown	6 (20)
Cold‐cup base biopsy, *n* (%)	21 (70)
Solid tumour, *n* (%)	12 (40)
Papillary tumour, *n* (%)	21 (70)
Macroscopically clear, *n* (%)	28 (93)
Tumour Stage* (*n* = 28), *n* (%)	
pT2	1 (3.6)
pT1	7 (25.0)
pTa	14 (50.0)
Benign	6 (21.4)
NICE Risk Score* (*n* = 28), *n* (%)	
High	9 (32.1)
Intermediate	9 (32.1)
Low	4 (14.3)
Not applicable	6 (21.4)
EAU Risk Score* (*n* = 28), *n* (%)	
Very high	3 (10.7)
High	6 (21.4)
Intermediate	13 (46.4)
Not applicable	6 (21.4)

AAA, abdominal aortic aneurysm; NICE, National Institute for Health; TIA, transient ischaemic attack. *Tumour stage, NICE and EAU risk group analyses were performed in 28 patients, excluding one case of metastatic prostate adenocarcinoma and one case of metastatic breast carcinoma. Not applicable comprised benign pathology, including four cases of cystitis cystica, one case of inflammation, and one case of non‐keratinising squamous metaplasia.

Detrusor muscle was absent on final histopathology in 13% (four of 30) of the TURBT specimens. For tumour stage and risk stratification analyses, two cases of metastatic non‐urothelial malignancy were excluded (*n* = 28), as reflected in Table 1. T‐stage was: pathological T‐stage (pT)2 in 3.6% (one of 28), pT1 in 25% (seven of 28), and pTa in 50% (14 of 28). Tumour grade was low in 25% (seven of 28) and high in 50% (14 of 28), respectively. The EAU risk groups were intermediate in 46% (13 of 28), high in 21% (six of 28), and very high in 11% (three of 28), respectively.

Once the learning curve was achieved, the median (IQR) time for FCM DM status was 5.39 (4.95–5.39) min. The median (IQR) time from TURBT to the final histopathology report was 11 (7–13.8) days.

### Primary Objective

**Fig. 1:**
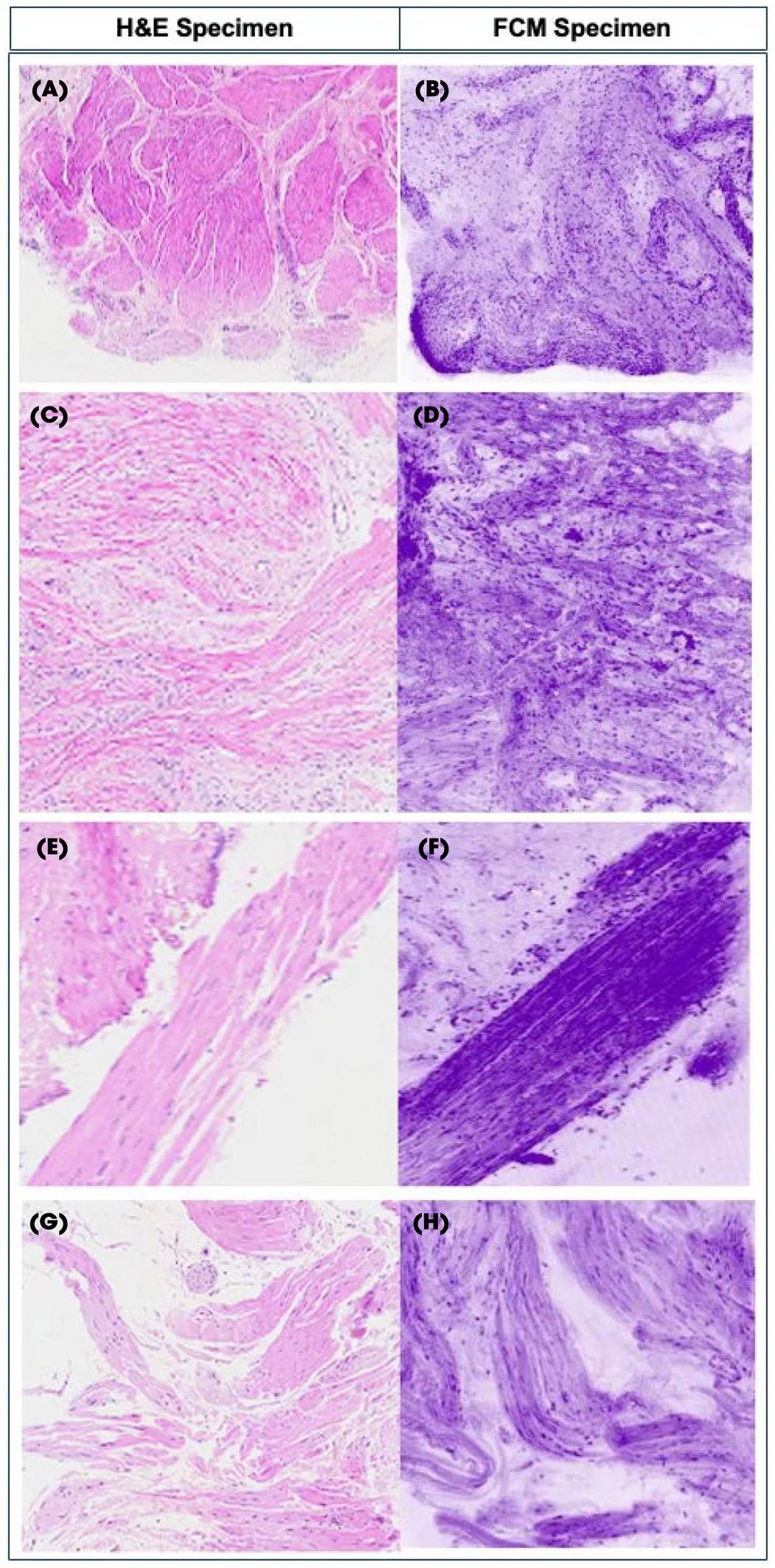
Comparison of paired haematoxylin and eosin (H&E) histopathology images and FCM. Comparison of paired H&E histopathology images and FCM images (*n* = 4) of deep bladder tissue obtained from TURBT with DM present in all images. (**A**, **B**) ‘A’ represents an H&E image of a deep resection of a bladder tumour, with transverse and longitudinal muscle bundles, and ‘B’ represents the corresponding FCM image, with a similar distribution of muscle fibres; (**C**, **D**) ‘C’ represents an H&E image of intersecting muscle fibres with intervening stroma, and ‘D’ represents the FCM image demonstrating similar features; (**E**, **F**) ‘E’ represents an H&E image of transverse muscle fibres, with fibrous stroma at the top left corner, which is similarly represented by the corresponding FCM image ‘F’; (**G**, **H**) ‘G’ represents a H&E image of transverse and longitudinal intersecting muscle fibres, which is similarly represented by the FCM image ‘H’.

Following blinded uro‐pathologist review of FCM images, all patients on study had sufficient image quality to interpret DM status (100% [30 of 30]; Fig. 1).

### Diagnostic Performance and Inter‐Observer Variability of FCM

The exploratory diagnostic performance characteristics are reported in Table 2. For FCM DM status, the sensitivity, specificity, PPV, and NPV were 42% (95% CI 26–61%), 100% (95% CI 51–100%), 100% (95% CI 74–100%), and 21% (95% CI 9–43%), respectively.

**Table 2:** Performance of FCM on a per‐patient level.

(A)			
	Final pathology		Total
	DM present	DM not present	
FCM			
Present	11	0	11
Not present	15	4	19
Total	26	4	30

(B)	
Metric	Estimate (95% CI*), %
True prevalence	87 (70–95)
Apparent prevalence	37 (22–54)
Sensitivity	42 (26–61)
Specificity	100 (51–100)
PPV	100 (74–100)
NPV	21 (9–43)
% Correctly classified	50 (33–67)

(A) the 2 × 2 contingency table shows the raw counts for tissues/patients judged to have DM or not by digital FCM compared to pathology, out of 30. (B) Sensitivity, specificity, PPV and NPV of digital FCM for the detection of DM compared to traditional haematoxylin and eosin histopathology as the reference standard.

* Wilson CIs.

The proportion of agreement (simple matching coefficient) between the uro‐pathologist and urologist was 0.87 (95% CI 0.75–0.99), with proportion of positive agreement of 0.83 (95% CI 0.67–0.99). Inter‐observer agreement was ‘high’ with a Kappa score of κ = 0.72 (95% CI 0.47–0.97; *P* < 0.001; [Table S1]).

### Discordant Analysis

Of the 15 discordant cases, where DM was present on final histopathology but absent on uro‐pathologists review of FCM images, 40% (six of 15) of patients had ‘scant’ or ‘fragments of DM’, and a further 60% (nine of 15) had clear DM present. When a representative definition was used treating scant or fragments of DM as no muscle, the sensitivity, specificity, PPV, and NPV were 45% (95% CI 27–65%), 88% (95% CI 53–98%), 91% (95% CI 62–98%), and 37% (95% CI 19–59%), respectively (Table S2).

### Exploratory Analysis of Tumour Grade and Stage Using FCM

Urothelial carcinoma was identified on FCM uro‐pathologist review in 53% (16 of 30) of cases, with no tumour identified in 43% (13 of 30), and interpretation not possible in 3.3% (one of 30) (Table 3). On FCM uro‐pathologist review, tumour grade was reported as high grade in 20% (six of 30) and low grade in 30% (nine of 30). In the remaining cases, tumour was not identified (43% [13 of 30]) or grade could not be assessed (6.7% [two of 30]).

**Table 3:** Tumour characteristics comparing the final pathology with interpretation of the FCM images.

Tumour characteristics	Overall histopathology, *n* (%)	Paired FCM specimen histopathology, *n* (%)	FCM specimen interpretation, *n* (%)
**Tumour type**			
Urothelial carcinoma	21 (70)	9 (30.0)	16 (53.3)
Metastatic breast cancer	1 (3.3)	1 (3.3)	0 (0)
Metastatic prostate adenocarcinoma	1 (3.3)	1 (3.3)	0 (0)
Squamous cell carcinoma	1 (3.3)	1 (3.3)	0 (0)
Cystitis cystica	4 (13.3)	4 (13.3)	0 (0)
Non‐keratinising squamous metaplasia	1 (3.3)	1 (3.3)	0 (0)
Inflammation	1 (3.3)	0 (0)	0 (0)
No tumour	0 (0)	13 (43.3)	13 (43.3)
Unable to assess	0 (0)	0 (0)	1 (3.3)
**Carcinoma *in situ* (CIS)**			
Yes	4 (13.3)	2 (6.7)	0 (0)
No	22 (73.3)	20 (66.7)	1 (3.3)
Unable to assess	3 (10)	8 (26.7)	23 (76.7)
N/A	1 (3.3)	0 (0)	6 (20)
**Lymphovascular invasion (LVI)**			
Yes	8 (26.7)	3 (10.0)	0 (0)
No	21 (70)	15 (50.0)	1 (3.3)
Unable to assess	1 (3.3)	0 (0)	22 (73.3)
N/A	0 (0)	12 (40.0)	7 (23.3)
**WHO 1973 Grade**			
Grade 2	9 (30)	5 (16.7)	12 (40)
Grade 2/Grade 3	6 (20)	1 (3.3)	0 (0)
Grade 3	6 (20)	2 (6.7)	1 (3.3)
No tumour or N/A	9 (30)	21 (70.0)	13 (43.3)
Unable to assess	0 (0)	1 (3.3)	4 (13.3)
**WHO 2004**			
LG	7 (23.3)	2 (6.7)	9 (30)
HG	14 (46.7)	6 (20.0)	6 (20)
No tumour or N/A	9 (30)	21 (70.0)	13 (43.3)
Unable to assess	0 (0)	1 (3.3)	2 (6.7)

N/A, not applicable; LG, low grade; HG, high grade. Overall histopathology refers to the standard formalin‐fixed histopathological assessment of all TURBT specimens received. Paired specimen histopathology refers to the histopathological assessment of the same tissue scanned (highlighted in orange) with FCM technology to enable comparison.

On final histopathological assessment of the paired FCM specimens, tumour grade was high grade in 46.7% (14 of 30), low grade in 23.3% (seven of 30), and benign or not applicable in 30% (nine of 30), respectively.

## Discussion

### Summary of Main Results

To our knowledge, this is the first blinded paired‐cohort feasibility study of FCM evaluating intraoperative assessment of DM. We reported excellent intraoperative image acquisition and high specificity for the detection of DM. Whilst the study was not powered to interrogate diagnostic performance, the sensitivity and NPV were lower than expected. These findings have important implications for current and future clinical utility. As it stands, whilst the presence of DM identified on FCM was reliably confirmed on final histopathology, the absence of DM on FCM could not be used to confidently exclude its presence within the specimen. Our present results therefore limit the utility of FCM as a stand‐alone decision tool to ‘rule out’ DM during primary TURBT.

We recognise that an appropriately powered diagnostic accuracy study is required to confirm where the technology is likely to be best utilised within the TURBT pathway. If diagnostic accuracy results are replicated the technology would likely be limited to a small group of patients with pTa high‐grade disease, in whom absence of DM on standard resection might prompt immediate additional resection at primary TURBT. However, should a substantially higher NPV be achieved, the test may serve as a rule out for DM at first resection, with practice changing clinical implications.

### Discordant Cases and Limitation of FCM DM Assessment

The discordant analyses showed that 40% of the patients with absent DM on FCM imaging had non‐representative DM on final pathology, with only scant DM present. In such cases, DM fibres likely lay deeper within the acquired tissue and therefore fall outside the *en face* surface captured by FCM, which scans only the exposed tissue plane. Additional technical factors, such as diathermy artefact, uneven specimen thickness, or suboptimal tissue scanner contact may also have accounted for reduced visibility of deeper muscle fibres. It is possible that *en bloc* resection approaches may improve special orientation and potentially reduce diathermy artefact at time of FCM.

### Comparison with Existing Literature and Future Clinical Utility

We were unable to identify prior blinded clinical trial evidence supporting FCM in bladder cancer. The Uleri et al. [19] case series of 48 patients undergoing FCM used solely *en bloc* TURBT, DM was present in 81% (39 of 48; 95% CI 68–90%). As with this study the time to produce FCM images was fast (<5 min) in contrast with the median time taken to obtain the final histopathological report of 11 days in our cohort. In this unblinded series uro‐pathologists reported a significantly higher sensitivity of 88% but lower specificity of 53% for DM, which we were unable to replicate under blinded trial conditions. More recently, another unblinded study scanning all TURBT tissue in 14 patients interpreted by two uro‐pathologists also demonstrated more favourable diagnostic performance than our study [20].

Together, these studies provide proof‐of‐concept for intraoperative FCM of DM at TURBT. However, substantial methodological differences that include the unblinded study designs, imaging of resected surfaces rather than a separate paired deep tumour‐base specimens, reader expertise, and use of *en bloc* techniques limit meaningful comparison of the diagnostic accuracy performance to our presented prospective blinded study.

Numerous randomised studies have consistently reported that *en bloc* resection leads to higher rates of DM in TURBT specimens [9, 21]. The higher rates of DM in our study where piecemeal resection was used are likely due to the Hawthorne effect, also known as the ‘observer effect’, where surgeons modify behaviour when under observed trial conditions [22, 23]. When applied to TURBT DM, rates improve in both control and experimental arms when compared to real‐world studies (30–60%) [4, 9, 24]. In our unit the DM rate was 12% higher on study compared to the immediately preceding cases (87% vs 76%).

Prior research focus has been on surgeon training and intraoperative checklists to possibly improve DM rates [25, 26]. However, a large cluster‐randomised trial (RESECT; ClinicalTrials.gov identifier: NCT05154084) utilising this approach did not replicate improvement DM rates in the intervention arm, and notably there was also no difference in early recurrence‐free survival [26]. In contrast to attempting to overcome surgeon behavioural factors, intraoperative digital pathology offers a potential replicable and durable solution to maintain consistent DM status over time, contingent on future improvements in diagnostic performance.

Second resections carry substantial heath‐economic costs and negatively impact patients’ quality of life [10, 11]. In the UK, the PHOTO randomised study (ISRCTN84013636) estimated the mean cost of a TURBT was £11 934 (€13 700) per patient on pathway [10]. With ~23 252 TURBTs performed annually and an estimated 6900 lacking DM, routine re‐resection in all patients would cost up to £82 million (€94 million) each year.

Our findings of strong agreement between the uro‐pathologist and urologist highlight the potential utility of bedside reporting, enabling immediate decisions regarding re‐resection guided by the urologist alone. This approach offers significant health‐economic benefits by eliminating the need for costly real‐time uro‐pathology interpretation of FCM images [27]. Furthermore, our feasibility study demonstrates that urologists can determine DM status within 5 min. This could be further improved by using artificial intelligence to ‘traffic light’ report DM status, as has been successfully performed in automated pathology reporting of *ex vivo* microscopy imaging at the time of Mohs surgical removal of skin cancer [28].

### Limitations

A key limitation of this study is the small sample size, which was intentionally chosen to assess initial feasibility. Conducting this pilot in a single tertiary referral centre, and the ‘observer effect’, resulted in a higher prevalence of DM in TURBT specimens (87%) than prior retrospective studies and may not reflect routine urological practice.

Overall, our findings support that FCM should not currently be used to guide intraoperative decision‐making for DM assessment outside of research settings.

## Conclusion

Fluorescence confocal microscopy is feasible for intraoperative detection of DM at the time of primary TURBT. The technology is rapid and can be reported by bedside urologists. Future appropriately powered diagnostic accuracy studies are required to confirm the diagnostic performance and role in guiding surgical decision‐making for immediate re‐resection.

## Funding

The study funders (The Urology Foundation and Penguins Against Cancer Charity) had no role in the study design, data collection, data analysis, data interpretation, or writing of the report.

## Author Contributions

Mr Martin J. Connor was the Principal Investigator. Mr Mathias Winkler was Chief Investigator. Dr Archana Gopalakrishnan was the clinical research fellow. Prof. Mona El‐Bahrawy postulated the study hypothesis and conducted the proof‐of‐concept.

**Trial co‐investigators**: Nikhil Mayor, Eva Bolton, Sami Hamid, Sai Kalpitha Reddy, Tayla Perreau, Eslam Maher, Alexander Light, Samuel Morris, Jiten Jaipura, Mariana Bertoncelli Tanaka, Norma Gibbons, Archie Hughes‐Hallett, Hashim U. Ahmed, Rakesh Heer, Andrew Smith, David Hrouda, Anna Silvanto.

**Study statistician**: Eslam Maher.

**Clinical trial manager**: Tayla Perreau.

All authors collated and interpreted data, and edited, reviewed, and approved the final report.

## Disclosure of Interests

Dr Martin J. Connor receives funding from the UK NIHR, NIHR Imperial Biomedical Research Centre (BRC), Prostate Cancer UK (PCUK), University College London Hospitals (UCLH) Charity, Penguins Against Cancer Charity and The Urology Foundation (TUF) for his research into urological cancers. Dr Nikhil Mayor receives funding from TUF, and the UK NIHR for his research into urological cancers. Dr Alex Light receives funding from TUF and the NIHR for his research into urological cancers. Prof. Rakesh Heer receives funding from the NIHR and PCUK for his research into urological cancers. Prof. Hashim U. Ahmed receives infrastructure support from the NIHR Imperial Biomedical Research Centre and Imperial College Experimental Cancer Medicine Centre; receives core funding from the UK NIHR Imperial BRC, the Wellcome Trust, the UK NIHR, the UK Medical Research Council, Cancer Research UK, PCUK, TUF, Imperial Health Charity, UCLH Charity, Imperial Prostate Foundation, Sonablate, and previously BMA Foundation, Trod Medical, Sophiris Biocorp and Angiodynamics for trial work. He has received travel allowance from Sonablate for conference attendance; was a paid consultant for Sophiris Biocorp and Sonablate; and is a proctor for Rezum treatment and cryotherapy for Boston Scientific and a paid proctor for high‐intensity focused ultrasound (HIFU) by Sonablate. He has received speaker honoraria from Astellas, Janssen and Varian and is on the medical advisory boards for Francis Medical and Janssen. Dr Mathias Winkler receives funding from The John Black Charitable Foundation, Penguins Against Cancer Charity and TUF for his research into urological cancers. The remaining authors have no conflicts of interest.

## Supporting information


**Data S1.** IB1 LASERComplete Study Protocol.

**Fig. S1.** The IB1‐LaserComplete study schema.
**Fig. S2.** Intraoperative process of completing FCM acquisition.
**Fig. S3.** Consolidated Standards of Reporting Trials (CONSORT) diagram depicting patient participation in the trial.
**Table S1.** Inter‐observer agreement between urologist and uro‐pathologist reporting FCM.
**Table S2.** Performance of FCM on a per‐patient level using representative muscle definition.

## Data Availability

The data that support the findings of this study are available on request from the corresponding author. The data are not publicly available due to privacy or ethical restrictions. Data Sharing: permission from the Executive/Writing Committee is necessary prior to disclosing any information relative to this study outside of the Trial Steering Committee. Any request by site investigators or other collaborators to access the study dataset must be formally reviewed by the Trial Management Group.

## References

[1] Siegel RL , Kratzer TB , Giaquinto AN , Sung H , Jemal A . Cancer statistics, 2025. CA Cancer J Clin 2025; 75: 10–45 39817679 10.3322/caac.21871 PMC11745215

[2] Ippoliti S , Bhatt NR , Ilie CP . Transurethral resection of bladder tumour (TURBT) as a day‐case: a real‐world practice and patients' perspective from a district general hospital (DGH). Urol J 2023; 90: 68–74 10.1177/03915603221110177 35819224

[3] Herr HW . The value of a second transurethral resection in evaluating patients with bladder tumors. J Urol 1999; 162: 74–76 10379743 10.1097/00005392-199907000-00018

[4] Maruniak NA , Takezawa K , Murphy WM . Accurate pathological staging of urothelial neoplasms requires better cystoscopic sampling. J Urol 2002; 167: 2404–2407 11992046

[5] Phull M , Begum H , John JB et al. Potential carbon savings with day‐case compared to inpatient transurethral resection of bladder tumour surgery in England: a retrospective observational study using administrative data. Eur Urol Open Sci 2023; 52: 44–50 37284039 10.1016/j.euros.2023.03.007 PMC10240513

[6] Mariappan P , Zachou A , Grigor KM . Detrusor muscle in the first, apparently complete transurethral resection of bladder tumour specimen is a surrogate marker of resection quality, predicts risk of early recurrence, and is dependent on operator experience. Eur Urol 2010; 57: 843–849 19524354 10.1016/j.eururo.2009.05.047

[7] Sylvester RJ , Van Der Meijden AP , Oosterlinck W et al. Predicting recurrence and progression in individual patients with stage ta T1 bladder cancer using EORTC risk tables: a combined analysis of 2596 patients from seven EORTC trials. Eur Urol 2006; 49: 466–477 16442208 10.1016/j.eururo.2005.12.031

[8] European Urology Association . EAU Guidelines on Non‐Muscle‐Invasive Bladder Cancer (TaT1 and CIS). Arnhem, the Netherlands: European Urology Association, 2023

[9] Gallioli A , Diana P , Fontana M et al. En bloc versus conventional transurethral resection of bladder tumors: a single‐center prospective randomized noninferiority trial. Eur Urol Oncol 2022; 5: 440–448 35618567 10.1016/j.euo.2022.05.001

[10] Yu G , Rice S , Heer R et al. Photodynamic diagnosis‐guided transurethral resection of bladder tumour in participants with a first suspected diagnosis of intermediate‐or high‐risk non–muscle‐invasive bladder cancer: cost‐effectiveness analysis alongside a randomised controlled trial. Eur Urol Open Sci 2023; 53: 67–77 37441343 10.1016/j.euros.2023.05.003 PMC10334235

[11] Zhang JH , Joyce DD , Shan Y et al. National complication and cost burden of transurethral resection of bladder tumor for bladder cancer. 2025 10.1016/j.urolonc.2025.03.004 PMC13572987 40274464

[12] Ongaro L , Rossin G , Biasatti A et al. Fluorescence confocal microscopy in urological malignancies: current applications and future perspectives. Life 2023; 13: 2301 38137902 10.3390/life13122301 PMC10744992

[13] Malvehy J , Pérez‐Anker J , Toll A et al. Ex vivo confocal microscopy: revolution in fast pathology in dermatology. Br J Dermatol 2020; 183: 1011–1025 32134506 10.1111/bjd.19017

[14] Villard A , Breuskin I , Casiraghi O et al. Confocal laser endomicroscopy and confocal microscopy for head and neck cancer imaging: recent updates and future perspectives. Oral Oncol 2022; 127: 105826 35316771 10.1016/j.oraloncology.2022.105826

[15] Mayor N , Light A , Silvanto A et al. IP8‐FLUORESCE: a prospective paired cohort study evaluating the diagnostic accuracy of fluorescence confocal microscopy for real‐time assessment of surgical margins in radical prostatectomy. Eur Urol 2025; 89: 223–232 41076406 10.1016/j.eururo.2025.09.4171

[16] Connor MJ , Gopalakrishnan A , Smith A et al. Ip11‐08 can ex vivo fluorescence confocal microscopy provide immediate bladder tumour pathology results at time of transurethral resection of bladder tumour (turbt)? J Urol 2025; 213(5S): e559

[17] Viechtbauer W , Smits L , Kotz D et al. A simple formula for the calculation of sample size in pilot studies. J Clin Epidemiol 2015; 68: 1375–1379 26146089 10.1016/j.jclinepi.2015.04.014

[18] Cohen JF , Korevaar DA , Altman DG et al. STARD 2015 guidelines for reporting diagnostic accuracy studies: explanation and elaboration. BMJ Open 2016; 6: e012799 10.1136/bmjopen-2016-012799 PMC5128957 28137831

[19] Buhas BA , Uleri A , Katzendorn O et al. Transition to a digital perioperative pathway for robot‐assisted radical prostatectomy: impact on patient outcomes and adherence. BJU Int 2025; 137: 473–479 41327902 10.1111/bju.70102

[20] Soputro NA , Wang L , Daher K et al. Intraoperative evaluation of detrusor muscle during transurethral resection of bladder tumour using confocal fluorescent microscopy. BJU Int 2026; 138: S49–S55 41672610 10.1111/bju.70165

[21] Hashem A , Mosbah A , El‐Tabey NA et al. Holmium laser en‐bloc resection versus conventional transurethral resection of bladder tumors for treatment of non–muscle‐invasive bladder cancer: a randomized clinical trial. Eur Urol Focus 2021; 7: 1035–1043 33386289 10.1016/j.euf.2020.12.003

[22] Pande A , Ghosh S . The hawthorne effect: quality and outcomes in neurosurgery. In Complications in Neurosurgery. Cham, Switzerland: Springer Nature Switzerland AG, 2023: 207–216 10.1007/978-3-030-12887-6_25 37548741

[23] Das A , Cohen JE , Ko OS et al. Surgeon scorecards improve muscle sampling on transurethral resection of bladder tumor and recurrence outcomes in patients with nonmuscle invasive bladder cancer. J Urol 2021; 205: 693–700 33021430 10.1097/JU.0000000000001372

[24] Struck JP , Moharam N , Leitenberger A et al. An international multicentre randomised controlled trial of en bloc resection of bladder tumour vs conventional transurethral resection of bladder tumour: first results of the en bloc resection of urothelium carcinoma of the bladder (EBRUC) II trial. BJU Int 2025; 135: 446–455 39462182 10.1111/bju.16543 PMC11842884

[25] Taoka R , Tsunemori H , Matsuoka Y et al. Use of surgical checklist during transurethral resection increases detrusor muscle collection rate and improves recurrence‐free survival in patients with non‐muscle‐invasive bladder cancer. Int J Urol 2021; 28: 727–732 33742465 10.1111/iju.14548

[26] Gallagher KM , MacLennan S , Bhatt NR , Clement KD , Zimmermann E . RESECT: a randomised controlled trial of audit and feedback in non‐muscle‐invasive bladder cancer surgery. Eur Urol 2025; 89: 355–365 41444076 10.1016/j.eururo.2025.09.4174

[27] Tang J , Ranasinghe W , Cheng J et al. Utility of routine intraoperative ureteral frozen section analysis at radical cystectomy: outcomes from a regional australian center. Curr Urol 2019; 12: 70–73 31114463 10.1159/000489422 PMC6504799

[28] Combalia M , Garcia S , Malvehy J et al. Deep learning automated pathology in ex vivo microscopy. Biomed Opt Express 2021; 12: 3103–3116 34221648 10.1364/BOE.422168 PMC8221965

