# Supplementary material for: Immediate intraoperative detrusor muscle status at primary transurethral resection of bladder tumour: a prospective, blinded, paired cohort, feasibility study using fluorescence confocal microscopy (IB1‐LaserComplete study)
Source: BJU Int. 2026 Aug 13;138(4):705–13. doi: 10.1111/bju.70395 (PMC13550801; doi:10.1111/bju.70395)
Supplement: Supplementary file 1 [file BJU-138-705-s001.pdf]

Imperial College London

NHS Imperial College Healthcare NHS Trust

Clinical Study Protocol

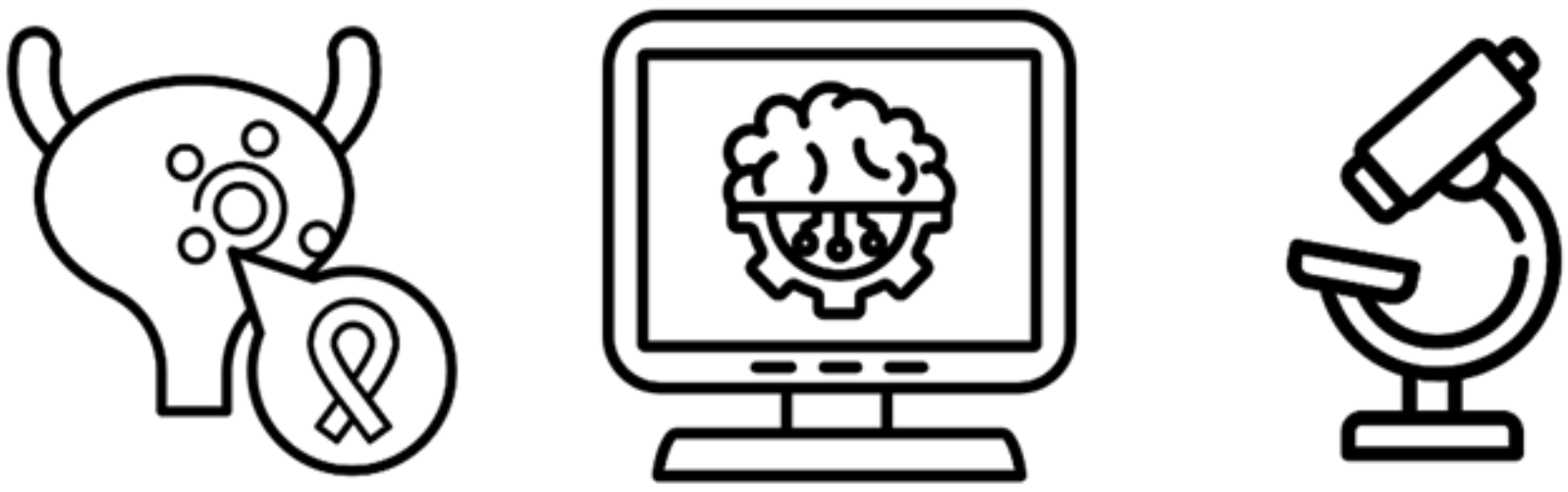

IB1-LaserCOMPLETE

Short Study Title

Imperial Bladder 1 (**IB1**) - Fluorescence **CO**nfocal **M**icroscopy for ra**P**id evaluation of detrusor muscle at primary transurethra**L** rs**E**ct**T**ion of bladd**E**r tumours

Fully Study Title

A prospective feasibility study to assess ex vivo real-time analysis of detrusor muscle status at time of primary transurethral resection of bladder tumour (TURBT) using fluorescence confocal microscopy

V1.0 4th July 2024

MAIN SPONSOR: Imperial College Healthcare NHS Trust

FUNDERS: NONE

ICHTB HTA licence: 12275

REC approval: 22/WA/0214

Protocol authorised by:

Name & Role	Date	Signature
Mr. Mathias Winkler<br>Chief Investigator	04/07/2024	
Mr. Martin J Connor;<br>Principal Investigator	04/07/2024	Martin Connor Digitally signed by Martin Connor Date: 2024.07.16 16:29:24 +01'00'

© Imperial College of Science, Technology and Medicine

Trial Management Group

Chief Investigator: Mr. Mathias Winkler
Investigator's Title: Visiting Reader and Consultant Urologist
Department & Address: Dept of Urology, CXH, ICNHT and ICL
Post code: W6 8RF
Phone: 07957396866
Email: mathias.winkler@nhs.net

Principal Investigator: Mr Martin J. Connor
Investigator's Title: NIHR Academic Clinical Lecturer
Department & Address: Dept of Urology, CXH, ICNHT and ICL
Post code: W6 8RF
Phone: 07986223438
Email: martin.connor2@nhs.net

Co-Investigators: Prof. Mona El-Bahrawy
Investigator's Title: Professor of Practice and Consultant Histopathologist, Imperial College London, UK
Department & Address: Department of Histopathology, 1st Floor, Hammersmith Hospital, Hammersmith Campus, CXH, ICNHT and ICL
Post code: W12 0NN
Email: m.elbahrawy@imperial.ac.uk

Co-investigators: Ms Eva Bolton
Investigator's Title: Consultant Urological Surgeon
Department & Address: Dept of Urology, CXH, ICNHT
Email: eva.bolton@nhs.net

Co-investigators: Mr Nikhil Mayor
Investigator's Title: Academic Clinical Fellow (ST3)
Department & Address: Dept of Urology, CXH, ICNHT and ICL
Email: nikhil.mayor@nhs.net

Co-investigators: Dr Archana Gopalakrishnan
Investigator's Title: Doctoral Clinical Research Fellow (CRF)
Department & Address: Dept of Urology, CXH, ICNHT and ICL
Email: archana.gopalakrishnan@imperial.ac.uk

Co-investigators: Mr David Hrouda
Investigator's Title: Consultant Urological Surgeon
Department & Address: Dept of Urology, CXH, ICNHT
Email: d.hrouda@nhs.net

Co-investigators: Ms Norma Gibbons
Investigator's Title: Consultant Urological Surgeon
Department & Address: Dept of Urology, CXH, ICNHT
Email: normagibbons@nhs.net

© Imperial College of Science, Technology and Medicine

Co-investigators: Prof. Rakesh Heer
Investigator's Title: Chair in Urology
Department & Address: Dept of Urology, CXH, ICNHT and ICL
Post code: W6 8RF
Email: r.heer@imperial.ac.uk

Co-investigators: Mr Archie Hughes-Hallett
Investigator's Title: Consultant Urological Surgeon
Department & Address: Dept of Urology, CXH, ICNHT and ICL
Email: ahughes-hallett@nhs.net

Statistician: *To be appointed*

Study Management:
Dr. Xiaomeng Li Clinical Trial Manager (ECMC)
Division of Surgery, Department of Surgery and Cancer, Imperial College London
xiaomeng.li@imperial.ac.uk

For general queries, supply of study documentation, and collection of data, please contact:

Study Coordinator: **Mr Martin J Connor & Ms Emma Cullen**
Address: Division of Surgery and Cancer
Hammersmith Hospital
Du Cane Road
W12 0HS
Tel: 07986223438
E-mail: m.connor@imperial.ac.uk & e.cullen@imperial.ac.uk

Clinical Queries

Clinical queries should be directed to Mr Martin Connor who will direct the query to the appropriate person

Sponsor

Imperial College Healthcare NHS Trust is the main research Sponsor for this study. For further information regarding the sponsorship conditions, please contact the Head of Regulatory Compliance at:

Research Governance and Integrity Team
Imperial College London and Imperial College Healthcare NHS Trust
Room 215, Level 2, Medical School Building
Norfolk Place
London, W2 1PG
Tel: **0207 594 1862**
Imperial College - Research Governance and Integrity Team (RGIT) Website

© Imperial College of Science, Technology and Medicine

This protocol describes the IB1-LASERComplete study and provides information about procedures for entering participants. Every care was taken in its drafting, but corrections or amendments may be necessary. These will be circulated to investigators in the study. Problems relating to this study should be referred, in the first instance, to the Chief Investigator.

This study will adhere to the principles outlined in the UK Policy Frame Work for Health and Social Care Research. It will be conducted in compliance with the protocol, the Data Protection Act and other regulatory requirements as appropriate.

© Imperial College of Science, Technology and Medicine

Table of Contents **Page No**

1. INTRODUCTION ... 8
1.1. LAY SUMMARY ... 8
1.2. BACKGROUND ... 8
1.3. RATIONALE FOR CURRENT STUDY ... 9
2. STUDY OBJECTIVES ... 10
3. STUDY DESIGN ... 10
3.1. STUDY OUTCOME MEASURES ... 12
4. PARTICIPANT ENTRY ... 12
4.1. PRE-REGISTRATION EVALUATIONS ... 12
4.2. INCLUSION CRITERIA ... 12
4.3. EXCLUSION CRITERIA ... 13
4.4. WITHDRAWAL CRITERIA ... 13
5. ADVERSE EVENTS ... 13
5.1. DEFINITIONS ... 13
5.2. REPORTING PROCEDURES ... 14
6. ASSESSMENT AND FOLLOW-UP ... 14
7. STATISTICS AND DATA ANALYSIS ... 14
8. REGULATORY ISSUES ... 14
8.1. ETHICS APPROVAL ... 14
8.3. CONSENT ... 14
8.4. CONFIDENTIALITY ... 15
8.5. INDEMNITY ... 15
8.6. SPONSOR ... 15
8.7. FUNDING ... 15
9. STUDY MANAGEMENT ... 15
10. PUBLICATION POLICY ... 15
11. GANNT Chart ... 16
12. REFERENCES ... 17

© Imperial College of Science, Technology and Medicine

GLOSSARY OF ABBREVIATIONS

AE	Adverse effect
AUC	Area under the curve
BCC	Basal cell carcinoma
BCR	Biochemical recurrence
BRC	Biomedical Research Centre
CE	Conformité Europóene
CRF	Case report form
ECG	Electrocardiogram
FCM	Fluorescence confocal microscopy
H&E	Haematoxylin & eosin
HTA	Human Tissue Act
ICHTB	Imperial College Healthcare Tissue Bank
ISRCTN	International Standard Randomised Controlled Trial Number
ISUP	International Society of Urological Pathology
NHS	National Health Service
REC	Research ethics committee
TURBT	Transurethral Resection of Bladder Tumour
SAE	Serious adverse effect
SOP	Standard operating procedure
TMG	Trial management group
MMC	Mitomycin-C
BCG	Bacillus Calmette–Guérin
MIBC	Muscle-invasive bladder cancer
NMIBC	Non-muscle invasive bladder cancer
EAU	European Association of Urology
NICE	National Institute for Health and Care Excellence

KEYWORDS
Bladder cancer, transurethral resection of bladder tumour, non-muscle invasive bladder cancer (NMIBC), fluorescence confocal microscopy, surgical margins, novel technology, histopathology

STUDY SUMMARY

**TITLE**	A prospective feasibility study to assess ex vivo real-time analysis of detrusor muscle status at time of primary transurethral resection of bladder tumour (TURBT) using fluorescence confocal microscopy
**DESIGN**	Prospective, feasibility design (ex vivo)
**OBJECTIVES**	**Primary**

© Imperial College of Science, Technology and Medicine

To determine the feasibility of using fluorescence confocal microscopy to identify the presence of detrusor muscle in primary transurethral resection of bladder tumour (TURBT) specimens

Secondary

Establish a standard operating procedure for scanning fresh bladder tissue from primary TURBTs.

To assess the correlation of detrusor muscle detection between fluorescence confocal microscopy and standard of care histopathology within the feasibility studies limited number of patients

Generate initial pilot data to calculate an appropriately powered sample size for a follow on prospective, comparative, blinded multicentre cohort study to detect detrusor muscle in TURBT specimen against standard of care histopathology reporting

OUTCOME MEASURES

Primary

Feasibility will be determined by ability to obtain sufficient digital FCM for interpretation of detrusor muscle from TURBT samples.

Secondary

Agreement of digital FCM with the pathology report for detrusor presence on a specimen at a patient level. Sensitivity, specificity, positive and negative predictive value of digital FCM for detection of detrusor muscle with traditional H&E histopathology as the reference standard, on a per-patient basis.

Agreement between readers: a) two individual histopathologists b) histopathologist vs urologist (Cohen's kappa coefficient)

POPULATION

Patients undergoing initial or first transurethral resection of bladder tumour (TURBT) for suspected bladder cancer.

ELIGIBILITY

Inclusion Criteria

- Patients undergoing initial or first transurethral resection of bladder tumour (TURBT) for suspected bladder cancer.

Exclusion Criteria

- Radiological or clinical suspicion of muscle invasive bladder cancer (cT2-T4)
- Prior diagnosis of NMIBC or MIBC on prior resection
- Patients who do not consent for ex vivo tissue research through Imperial College Healthcare Tissue Bank (ICHTB).
- Patients enrolled in concurrent clinical trials requiring ex vivo tissue for research.

DURATION

The recruitment period will begin in July 2024 and run for a total of 6 months or until the required number of cases have been recruited.

© Imperial College of Science, Technology and Medicine

1. INTRODUCTION

1.1. LAY SUMMARY

Bladder cancer is the seventh most commonly diagnosed cancer and it is 3 to 4 times more common in men than in women[2]. Bladder cancer is first diagnosed by tissue obtained from a transurethral resection of bladder tumour (TURBT) operation. This involves passing a telescope into the bladder along the urethra (waterpipe) and removing bladder tumours (growths) using diathermy (electrical current) or laser energy.

In 7 in every 10 patients diagnosed with bladder cancer the cancer is present in the “superficial” layer of the bladder and does not grow deeper into the bladder muscle layer. Treatments for this form of superficial cancer such as bladder installations of chemotherapy or immunotherapy have been effective in reducing the chance of the bladder cancer recurring or progressing overtime.

Therefore, a key determinant of the correct treatment allocation following this operation is whether muscle was obtained to allow a pathologist to report the correct depth of cancer invasion.

Unfortunately, performing this operation can be challenging and in up to 30% of patients there will be no muscle present in the tissue obtained to make an accurate assessment. The knowledge of this will become available 1-2 weeks following the operation. In most patients the treating urologist will ask for the operation to be repeated to obtain this muscle sample. This has a significant impact upon patients health, quality of life, and a large financial burden on our healthcare service.

We are proposing to use a novel scanner known as “fluorescence confocal microscopy” that could scan and report acquired bladder tissue in the operating theatre “live” to determine if muscle is present, providing immediate feedback to the operating surgeon.

This technology has been used in other urological cancers such as prostate cancer to determine if prostate cancer has spread beyond the gland at time of prostate removal in real-time. However, it has never been used in this form of bladder cancer operation. If in our study this is proven to be possible, we plan to perform a larger practice changing study comparing this technology against traditional reporting.

1.2. BACKGROUND

Fluorescence confocal microscopy (FCM) is an imaging technique whereby digital images of fresh tissues are produced almost instantly, without the need for time-consuming fixation techniques[1]. It has been evaluated across several different organ systems and can accurately diagnose prostate cancer when compared to conventional histopathology[1].

Bladder cancer is the seventh most diagnosed cancer and it is 3 to 4 times more common in men than in women[2]. Bladder cancer is first diagnosed by tissue obtained from a transurethral resection of bladder tumour (TURBT) endoscopic operation. Over 75% of patients present with disease that is confined to the mucosa (pTa, CIS) or submucosa (pT1) or non-muscle invasive bladder cancer (NMIBC)[2,3]. High-risk NMIBC is defined by the National Institute for

© Imperial College of Science, Technology and Medicine

Health and Care Excellence (NICE) as any pT1aG3, any pT1(G2/3), pTis (Cis) or any variant urothelial carcinoma (e.g. micropapillary or nested variant)[3].

Effective treatments for NMIBC such as bladder instillations of Bacillus Calmette-Guérin (BCG) and Mitomycin-C reduce progression and recurrence[2,3]. A key determinant of correct allocation of treatment at the time of TURBT is whether detrusor muscle was obtained to allow accurate determination of pathological stage (depth of invasion)[2,4]. Prior studies have reported the absence of detrusor muscle in the specimen is associated with a significantly higher risk of residual disease, early recurrence, and tumour under-staging[4,5]. In contrast the presence of detrusor muscle in the specimen is considered a surrogate measure of the "resection quality" [2,4,5].

1.3. RATIONALE FOR CURRENT STUDY

Numerous studies have suggested that in the real world approximately 30% (15%-65%) of patients in this group do not have muscle in their first resection[4,6-8]. *National Institute for Health and Care Excellence* (NICE) and *European Association of Urology* (EAU) have issued strong guidance that newly-diagnosed patients without muscle in the specimen should undergo a repeat or "second-look" TURBT resection of tumour bearing areas to determine the exact stage and refine allocation of treatment[2,3].

In 2022, 23,252 TURBTs were performed in the United Kingdom, where the lowest NHS tariff is £1,725[9]. It is expected that approximately 6,975 (30%) will have no muscle in the specimen. A re-resection will be indicated in most of these cases. Fluorescence confocal microscopy (FCM) using the Histolog® scanner has the potential to ensure muscle is present at time of first resection with live feedback to the operating surgeon. This may avoid over 6,000 TURBTs annually, a potential direct cost saving of £6.9 million to the NHS.

To the best of the authors knowledge, intraoperative analysis of the presence of muscle in TURBT specimens using fluorescence confocal microscopy has not been reported previously beyond a case report (*n*=3)[10]. Puliatti and colleagues' examined three patients who underwent a TURBT using the Mavig 2500 VivaScope® FCM machine and the images sent remotely for reporting by a trained histopathologist[10]. All specimens also underwent blinded paired histopathology reporting of Hematoxylin & Eosin stained slides as per standard of care. It was not reported if the study patients were undergoing an initial primary resection or re-look/second TURBT or for recurrence of known NMIBC. The author's reported that in all three patients there was concordance with the final histopathological report. In two of these patients high-risk NMIBC was present[10].

Prior large prospective efforts in "confocal microscopy" have been limited to in-vivo "laser confocal microscopy" mapping of the bladder to identify areas or bladder tumour recurrence and direct the operating surgeon to resect areas of interest[11-15]. Regarding other urological malignancy there is a growing body of evidence for FCM in prostate cancer[1,16]. The TUF funded IP8-FLUORESCE (ISRCTN21536411) is a prospective paired-cohort confirmatory study to assess the accuracy of digital fluorescence confocal microscopy for assessment of surgical margins in radical prostatectomy specimens and has completed accrual. The Histolog® scanner technology was utilised in this study successfully.

© Imperial College of Science, Technology and Medicine

What is FCM?

Analysing tissue samples using traditional light microscopy is limited by the thickness of the sample as out-of-focus light adds blur to images and reduces resolution. Confocal laser microscopy uses pinhole apertures in the microscope detector and provides a means of rejecting out-of-focus light, reducing blur and producing high-resolution imaging in thick tissue samples. Fluorescence confocal microscopy uses fluorochromes to increase the cell-to-stroma contrast. Depending on the laser wavelength, reflectance (488 nm) and fluorescence (785 nm) can be applied. Novel confocal microscopes allow visualisation with reflectance and fluorescence simultaneously, providing high resolution images in both modes separately and in combination (fusion mode). In fusion mode, the traditional greyscale is digitally enhanced to provide a two-colour scale which resembles haematoxylin and eosin (H&E) staining. FCM requires minimal tissue preparation (typically staining samples in Acridine Orange for 10 seconds then washing with saline) and can produce high-resolution digital images in less than a minute. The FCM machine is portable and requires only one square metre of space and can therefore be kept in the operating theatre or an office. Critically, the samples can be re-examined with conventional techniques after FCM as the fluorescent dye does not alter the specimen and tissue fixation is required. Images can be directly uploaded to electronic patient records for review by pathologists or trained urologists.

2. STUDY OBJECTIVES

Primary

To determine the feasibility of using fluorescence confocal microscopy to identify the presence of detrusor muscle in primary transurethral resection of bladder tumour (TURBT) specimens

Secondary

Establish a standard operating procedure for scanning fresh bladder tissue from primary TURBTs.

To assess the correlation of detrusor muscle detection between fluorescence confocal microscopy and standard of care histopathology within the feasibility studies limited number of patients

Generate initial pilot data to calculate an appropriately powered sample size for a follow on prospective, comparative, blinded multicentre cohort study to detect detrusor muscle in TURBT specimen against standard of care histopathology reporting

3. STUDY DESIGN

IB1-LASERComplete is a single-centre, unblinded, prospective, feasibility study. Recruitment will take place from one of the UK's largest designated urological cancer units and works in tandem with a dedicated Clinical Trials Unit. Over 150 TURBTs per year are performed by 4 surgeons.

© Imperial College of Science, Technology and Medicine

All consecutive patients undergoing primary TURBT and consent to Imperial College Healthcare Tissue Bank (ICHTB) will be included. Where there is radiological or clinical suspicion of muscle invasive bladder cancer (cT2-T4) or a prior diagnosis of NMIBC on prior resection these patients will be excluded.

The study is ex vivo and no deviations from standard of care will take place during the study.

3.1 Developing and feasibilty of developed standard operating procedure

Surgical technique

Standard monopolar or bipolar transurethral resection of bladder tumour (TURBT) will be performed by urologists who are part of imperial bladder cancer clinician group, as per existing local policy. Any resection pattern including "*en bloc*" and "*standard piecemeal*" resection will be accepted.

"LASERComplete" FCM procedure

Immediately after extraction of the specimen, digital FCM standardised images will be acquired from anterior and posterior surfaces of the largest specimen, up to three if present. An initial assessment for the presence of muscle will be made by a trained urologist (Mr. Martin J. Connor) and histopathologist (Prof. Mona El-Bahrawy). A deferred review of images and traditional histopathological assessment in line with local guidelines will be undertaken by a dedicated Uro-Pathologist. Both urologists and histopathologists need to pass the four-hour training module with the application specialist.

The exact SOP will be defined in an initial pilot phase of five cases. Digital FCM images will be acquired using the Histolog® Scanner (SamanTree Medical SA, Lausanne, Switzerland) which uses a single laser in fluorescence mode. A fast image mode (preview) provides an image of 1600x1600 pixels within 5 seconds and a second mode (acquire) offers a maximum-resolution image of 8000x8000 pixels within 50 seconds which can be used for histopathologic evaluation. A judgement will be made if scanned tissue sections need to be removed first or if the scanning field allows scanning the surface of an intact bladder tumour. We aim to utilise a mechanism of weighing down, stabilizing and compressing the intact specimen in order to avoid disruption of the specimen. The specimen will be immersed in a fluorescent agent (Histolog Dip, SamanTree Medical SA) for 10 seconds then rinsed in 0.9% saline. The specimen will be placed on the scanner tray producing an image 48x36 mm in one field of view with the external side facing down (en face). The preview image is acquired to make a technical judgement by the trained urologist or pathology technician on the completeness of the image, presence of excess fluid, and absence of air bubbles. If necessary, the specimen is repositioned before the final high-resolution digital image is acquired. The total procedure time will be recorded.

Histopathological analysis

After FCM images are acquired, tissues will be transferred fresh to the pathology department and treated with pigment solution, formalin-fixed for 24 hours and fully paraffin-embedded for routine diagnosis.

Outcome reporting

© Imperial College of Science, Technology and Medicine

The histopathology slides for each patient will be digitally scanned and uploaded to a secure cloud-based server alongside the FCM images. All files will be pseudoanonymised and the server locked until the recruitment target has been reached. At this stage, histopathology slides will be re-reported by an expert independent uro-pathologist using FCM images. They will be blinded to the histology report when making the research FCM report. The histopathologist will provide the overall muscle status on a per-patient level, as well as a report on a pT stage, presence or absence of carcinoma in-situ and grade (high / low)

The recruitment target is 5 patients for the initial phase, followed by 30 patients for the appraisal of the developed SOP. The study will run for 6 months or until the recruitment target is reached.

3.1. STUDY OUTCOME MEASURES

Primary

Feasibility will be determined by ability to obtain sufficient digital FCM for interpretation of detrusor muscle from TURBT samples

Secondary

Agreement of digital FCM with the pathology report for detrusor presence on a specimen at a patient level. Sensitivity, specificity, positive and negative predictive value of digital FCM for detection of detrusor muscle with traditional H&E histopathology as the reference standard, on a per-patient basis.

Agreement between readers: a) two individual histopathologists b) histopathologist vs urologist (Cohen's kappa coefficient)

4. PARTICIPANT ENTRY

4.1. PRE-REGISTRATION EVALUATIONS

All participants scheduled for TURBT undergo routine history taking, examination, diagnostic workup, and staging to ensure suitability for the procedure. All patients will undergo standard of care preoperative workup including renal and liver function testing, ECG, and anaesthetic review.

Due to the ex vivo design of the study, no screening visits are required. Further, patients will not have any contact, undergo any tests, or require any follow-up out of line with standard of care management other than the digital FCM scan intraoperatively.

4.2. INCLUSION CRITERIA

Any person over the age of 18 with a first or initial transurethral resection of bladder tumour (TURBT) at Imperial College Healthcare NHS Trust during the study period.

© Imperial College of Science, Technology and Medicine

4.3. EXCLUSION CRITERIA

Radiological or clinical suspicion of muscle invasive bladder cancer (cT2-T4)

Prior diagnosis of NMIBC or MIBC on prior resection

Patients who do not consent for ex vivo tissue research through Imperial College Healthcare Tissue Bank (ICHTB).

Patients enrolled in concurrent clinical trials requiring ex vivo tissue for research.

4.4. WITHDRAWAL CRITERIA

Patients may decide to opt out of IB1-LASERComplete at any time. This is entirely within their right to do so. Such cases will be reported to the research team so that no further data are entered onto the database. Data captured before consent was withdrawn will be used in the study, but no further data, beyond this date will be collected or used in any analysis. Reason for withdrawal must be recorded in the CRF/eCRF and medical records. We will continue to recruit patients until the target number for study is met.

5. ADVERSE EVENTS

5.1. DEFINITIONS

Adverse event will be reported in line with the Common Terminology Criteria for Adverse Events (CTCAE version 5.0)

Adverse Event (AE): any untoward medical occurrence in a patient or clinical study subject.

Serious Adverse Event (SAE): any untoward medical occurrence or effect that:

- **Results in death**
- **Is life-threatening** – *refers to an event in which the subject was at risk of death at the time of the event; it does not refer to an event which hypothetically might have caused death if it were more severe*
- **Requires hospitalisation, or prolongation of existing inpatients' hospitalisation**
- **Results in persistent or significant disability or incapacity**
- **Is a congenital anomaly or birth defect**

Medical judgement should be exercised in deciding whether an AE is serious in other situations. Important AEs that are not immediately life-threatening or do not result in death or hospitalisation but may jeopardise the subject or may require intervention to prevent one of the other outcomes listed in the definition above, should also be considered serious.

© Imperial College of Science, Technology and Medicine

5.2. REPORTING PROCEDURES

This is an ex vivo study. Any AEs that occur as a result of the RP procedure will be reported in line with usual clinical practice. It is not a requirement for the CI to be informed.

6. ASSESSMENT AND FOLLOW-UP

No follow-up or follow-up visits are required for this ex vivo study.

7. STATISTICS AND DATA ANALYSIS

Full details of planned statistical summaries and analyses will be outlined in a pre-defined, signed and dated statistical analysis plan before any analyses are performed. Any deviations from the analysis plan will be fully documented with reasons for doing so.

Data and all appropriate documentation will be stored for 10 years after the completion of the study.

8. REGULATORY ISSUES

8.1. ETHICS APPROVAL

ICHTB HTA licence: 12275. R24019: Ex vivo real-time analysis of detrusor muscle status at time of primary transurethral resection of bladder tumour (TURBT) using fluorescence confocal microscopy.

This study was granted ethical approval via the Imperial College Healthcare Tissue Bank (ICHTB) which is approved by Wales REC3 to release human material for research. The Human Tissue Authority (HTA) licence reference is 12275. Research ethics committee (REC) approval is granted under reference 22/WA/0214. ICHTB is supported by the National Institute for Health Research (NIHR) Biomedical Research Centre (BRC) based at Imperial College Healthcare NHS Trust and Imperial College London. Further information on Imperial's BRC can be found here:

https://imperialbrc.nihr.ac.uk/about-us/acknowledgement/.

The study will be conducted in accordance with the recommendations for physicians involved in research on human subjects adopted by the 18th World Medical Assembly, Helsinki 1964 and later revisions.

8.2. TRIAL REGISTRATION

The study will be registered on a trial database ISRCTN in accordance with requirements of the International Committee of Medical Journal Editors (ICMJE) regulations.

8.3. CONSENT

Consent for use of human tissue for medical research is part of the standard consent process for patients undergoing surgery or biopsy at Imperial College Healthcare NHS

© Imperial College of Science, Technology and Medicine

Trust. Patients receive an information leaflet via the ICHTB prior to signing consent. Only those patients who have signed this subsection of the surgical consent form will be included.

8.4. CONFIDENTIALITY

The investigator must ensure that the subject's confidentiality is maintained. On the CRF or other documents submitted to the Sponsors, subjects will be identified by a subject ID number only. Documents that are not submitted to the Sponsor (e.g., signed informed consent form) should be kept in a strictly confidential file by the investigator.
The investigator shall permit direct access to subjects' records and source document for the purposes of monitoring, auditing, or inspection by the Sponsor, authorised representatives of the Sponsor, NHS, Regulatory Authorities and REC.

The Chief Investigator will preserve the confidentiality of participants taking part in the study and is registered under the Data Protection Act.

8.5. INDEMNITY

Imperial College London holds negligent harm and non-negligent harm insurance policies which apply to this study/ Imperial College Healthcare NHS Trust holds standard NHS Hospital Indemnity and insurance cover with NHS Resolution for NHS Trusts in England, which apply to this study (delete as applicable)

8.6. SPONSOR

Imperial College Healthcare NHS Trust will act as the main Sponsor for this study.

8.7. FUNDING

No grant funding is currently available.

9. STUDY MANAGEMENT

A trial management group (TMG) including the Chief Investigator, co-investigators and key collaborators, trial statistician and trial manager. The TMG will be responsible for day-to-day conduct of the trial and operational issues. Details of membership, responsibilities and frequency of meetings will be defined separately. A lay person will be included.

10. PUBLICATION POLICY

Information concerning the study, patent applications, processes, scientific data or other pertinent information is confidential and remains the property of the Sponsor. The investigator may use this information for the purposes of the study only. It is understood by the investigator that the Sponsor will use information developed in this clinical study and, therefore, may disclose it as required to other clinical investigators. In order to allow the use of the information derived from this clinical study, the investigator understands that he/she has an obligation to provide complete test results and all data developed during this study to the Sponsor. Verbal or written discussion of results prior to study

© Imperial College of Science, Technology and Medicine

completion and full reporting should only be undertaken with written consent from the Sponsor. Therefore, all information obtained as a result of the study will be regarded as CONFIDENTIAL, at least until appropriate analysis and review by the investigator(s) are completed. A Clinical Study Report summarising the study results will be prepared and submitted to the ICHTB within a year of the end of study.

11. GANTT CHART

IB1-LASERComplete Trial	Year 1 2024				Year 2 2025			
	Q1	Q2	Q3	Q4	Q1	Q2	Q3	Q4
Study document preparation	X							
Development and set up	X	X						
Trial Open and Recruitment			X	X	X			
Follow-up			X	X	X	X		
Final data cleaning						X		
Database lock						X		
Statistical analysis							X	
Final report and manuscript								X
Archiving								X

© Imperial College of Science, Technology and Medicine

REFERENCES

1. Ongaro L, Rossin G, Biasatti A, et al. Fluorescence confocal microscopy in urological malignancies: Current applications and future perspectives. *Life*. 2023;13(12):2301.

2. European Urology Association. EAU guidelines on Non-muscle-invasive bladder cancer (TaT1 and CIS). . 2023.

3. National Institute for Health and Care Excellence. NICE guideline [NG2] bladder cancer: Diagnosis and management. . 2015.

4. Mariappan P, Zachou A, Grigor KM. Detrusor muscle in the first, apparently complete transurethral resection of bladder tumour specimen is a surrogate marker of resection quality, predicts risk of early recurrence, and is dependent on operator experience. *Eur Urol*. 2010;57(5):843-849.

5. Sylvester RJ, Van Der Meijden, Adrian PM, Oosterlinck W, et al. Predicting recurrence and progression in individual patients with stage ta T1 bladder cancer using EORTC risk tables: A combined analysis of 2596 patients from seven EORTC trials. *Eur Urol*. 2006;49(3):466-477.

6. Herr HW. The value of a second transurethral resection in evaluating patients with bladder tumors. *J Urol*. 1999;162(1):74-76.

7. Maruniak NA, Takezawa K, Murphy WM. Accurate pathological staging of urothelial neoplasms requires better cystoscopic sampling. *J Urol*. 2002;167(6):2404-2407.

8. Ippoliti S, Bhatt NR, Ilie CP. Transurethral resection of bladder tumour (TURBT) as a day-case: A real-world practice and patients' perspective from a district general hospital (DGH). *Urologia Journal*. 2023;90(1):68-74.

9. Phull M, Begum H, John JB, et al. Potential carbon savings with day-case compared to inpatient transurethral resection of bladder tumour surgery in england: A retrospective observational study using administrative data. *European Urology Open Science*. 2023;52:44-50.

10. Puliatti S, Eissa A, Bevilacqua L, et al. Ex vivo fluorescence confocal microscopy in the assessment of urothelial carcinoma grading in bladder and ureter: Our preliminary experience. *European Urology Supplements*. 2019;18(1):e226-e227.

11. Beji S, Wrist Lam G, Østergren PB, Toxvaerd A, Sønksen J, Fode M. Diagnostic value of probe-based confocal laser endomicroscopy versus conventional endoscopic biopsies of non-muscle invasive bladder tumors: A pilot study. *Scandinavian Journal of Urology*. 2021;55(1):36-40.

12. Lee JJ, Thomas I, Nolley R, Ferrari M, Brooks JD, Leppert JT. Biologic differences between peripheral and transition zone prostate cancer. *Prostate*. 2015;75(2):183-190.

13. Chang TC, Liu J, Hsiao ST, et al. Interobserver agreement of confocal laser endomicroscopy for bladder cancer. *Journal of endourology*. 2013;27(5):598-603.

14. Lopez-Beltran A, Blanca A, Cimadamore A, et al. T1 bladder carcinoma with variant histology: Pathological features and clinical significance. *Virchows Archiv*. 2022;480(5):989-998.

15. Marien A, Rock A, Maadarani KE, et al. Urothelial tumors and dual-band imaging: A new concept in confocal laser endomicroscopy. *Journal of endourology*. 2017;31(5):538-544.

16. Rocco B, Sighinolfi MC, Sandri M, et al. Digital biopsy with fluorescence confocal microscope for effective real-time diagnosis of prostate cancer: A prospective, comparative study. *European Urology Oncology*. 2021;4(5):784-791.

17. Sim J, Lewis M. The size of a pilot study for a clinical trial should be calculated in relation to considerations of precision and efficiency. *J Clin Epidemiol*. 2012;65(3):301-308.

18. Browne RH. On the use of a pilot sample for sample size determination. *Stat Med*. 1995;14(17):1933-1940.

© Imperial College of Science, Technology and Medicine
