# Supplementary material for: Immediate intraoperative detrusor muscle status at primary transurethral resection of bladder tumour: a prospective, blinded, paired cohort, feasibility study using fluorescence confocal microscopy (IB1‐LaserComplete study)
Source: BJU Int. 2026 Aug 13;138(4):705–13. doi: 10.1111/bju.70395 (PMC13550801; doi:10.1111/bju.70395)
Supplement: Supplementary file 2 [file BJU-138-705-s002.docx]

**Supplementary Material**

M.J. Connor *et al.* **Immediate intraoperative detrusor muscle status at primary transurethral resection of bladder tumour:** a prospective, paired cohort, feasibility study using fluorescence confocal microscopy (IB1-LaserComplete)

**Suppl. Figure 1: IB1-LaserComplete study schema**

**Suppl. Figure 2:** **Intraoperative process of completing FCM acquisition**

Legend: FCM images were acquired using the Histolog® Scanner (SamanTree SA, Switzerland) (A). The tissue sample is left for 10 seconds in the Histolog® Dip (acridine orange) (B), rinsed with saline (C) and excess liquid is drawn away by capillarity (D). The acquisition of a single-face, 48x36mm image of the tissue sample (E), with a maximum resolution of 8000x8000 pixels is acquired (F).

**Suppl. Figure 3:** Consort diagram depicting patient participation in the trial

**Suppl. Table 1. Inter-observer agreement between urologist and uro-pathologist reporting FCM**

|  | **FCM Pathologist** | |  |
| --- | --- | --- | --- |
|  | Present | Not present | **Total** |
| **FCM Urologist** |  |  |  |
| Present | 10 | 3 | **13** |
| Not present | 1 | 16 | **17** |
| **Total** | **11** | **19** | **30** |
|  | | | |

***Legend:*** Agreement between the urologist and the histopathologist on FCM interpretation: The cross-table below displays the classification of patients by the two readers using the binary scale in terms of counts. The unweighted Cohen’s Kappa coefficient is 0.72 (95% CI: 0.47 to 0.97), p < 0.001. This is interpreted as a “good” agreement (Altman 1990). The p-value is tested against the null of no agreement better than chance, i.e. kappa (k0) = 0. The proportion of agreement (simple matching coefficient) is 0.87 (95% CI: 0.75 to 0.99). The proportion of positive agreement is 0.83 (95% CI: 0.67 to 0.99).

**Suppl. Table 2. Performance of fluorescence confocal microscopy on a per-patient level using representative muscle definition**

**(i)**

|  | **Final Pathology** | |  |
| --- | --- | --- | --- |
|  | Present | Not present | **Total** |
| **FCM** |  |  |  |
| Present | 10 | 1 | **11** |
| Not present | 12 | 7 | **19** |
| **Total** | **22** | **8** | **30** |

**(ii)**

| Metric | Estimate | 0.95 CI^*^ |
| --- | --- | --- |
| True prevalence | 73% | (56, 86) |
| Apparent prevalence | 37% | (22, 54) |
| Sensitivity | 45% | (27, 65) |
| Specificity | 88% | (53, 98) |
| PPV | 91% | (62, 98) |
| NPV | 37% | (19, 59) |
| %Correctly classified | 57% | (39, 73) |

**Legend:** Representative detrusor muscle defined as detrusor muscle that is non-fragmented or scanty (i) the 2×2 contingency table below shows the raw counts for tissues/patients judged to have representative detrusor muscle or not by digital FCM compared to pathology, out of n=30. (ii) Sensitivity, specificity, positive and negative predictive values (PPV and NPV) of digital FCM for the detection of detrusor muscle compared to traditional H&E histopathology as the reference standard. *Wilson confidence intervals. FCM - fluorescence confocal microscopy.

**IB1-LASERComplete Histopathology Reporting Proforma**

**Patient MRN ID:** INSERT

**Histopathologist**: INSERT

**Date of TURBT:** *Trial team will insert following report*

**Specimen NHS Accession number:** *Trial team will insert following report*

| Are the FCM images provided sufficient for reporting the presence of detrusor muscle? | **Yes** / **No** |
| --- | --- |
| *If unable to report detrusor muscle status from provided images please state reason (free text)* |  |
| **Detrusor Muscle Status** | |
| Status of muscularis propria | **Present** / **Absent** |
| **Tumour Assessment** | |
| Is tumour present within the FCM images? | Yes / No / Unable to assess |
| Tumour type | ***Delete as appropriate***  No tumour  Unable to assess  Urothelial Carcinoma  Squamous Cell Carcinoma  Adenocarcinoma  Mullerian Type Tumour  Small Cell Neuroendocrine  Large Cell Neuroendocrine  Other: (free text) |
| WHO 1973 | ***Delete as appropriate***  No tumour present  Unable to assess  Grade 1  Grade 2  Grade 3 |
| WHO 2004 | **Delete as appropriate**  No tumour present  Unable to assess  Low Grade  High Grade |
| If squamous cell carcinoma or adenocarcinoma | ***Delete as appropriate***  N/A  Well differentiated  Moderately differentiated  Poorly differentiated |
| Maximum extent of tumour invasion | ***Delete as appropriate***  No tumour present  Unable to assess  Non-invasive papillary carcinoma  Tumour invades lamina propria (submucosa)  Tumour invades muscularis propria  Tumour invades prostatic ducts/acini  Tumour invades prostatic stroma |
| Associated CIS | ***Delete as appropriate***  Unable to assess  Yes (adjacent to tumour)  Yes (elsewhere)  No |
| Lymphovascular Invasion (LVI) | ***Delete as appropriate***  Unable to assess  Yes  No |

CRF V1.0, Imperial College London, 1^st^ Aug 2025

*Note: Modified from Standards and datasets for reporting cancers (Appendix D: Histopathology Reporting Proforma: transurethral specimens [biopsy or TUR]). PGD 040725 60 V4 Final; Royal College of Pathologists, July 2025.*
